# Generation of JC Polyoma Pseudovirus for High-Throughput Measurement of Neutralizing Antibodies

**DOI:** 10.3390/diagnostics14030311

**Published:** 2024-01-31

**Authors:** Mami Matsuda, Tian-Cheng Li, Akira Nakanishi, Kazuo Nakamichi, Makoto Saito, Tadaki Suzuki, Tomokazu Matsuura, Masamichi Muramatsu, Tetsuro Suzuki, Yoshiharu Miura, Ryosuke Suzuki

**Affiliations:** 1 Department of Virology II, National Institute of Infectious Diseases, Tokyo 208-0011, Japan; mami@niid.go.jp (M.M.); litc@niid.go.jp (T.-C.L.); muramatsu@niid.go.jp (M.M.); 2 Department of Genetic Engineering, Kindai University, Wakayama 649-6493, Japan; nakanishi@waka.kindai.ac.jp; 3 Department of Virology I, National Institute of Infectious Diseases, Tokyo 162-8640, Japan; nakamich@niid.go.jp; 4 Clinical Research Support Center, Tokyo Metropolitan Komagome Hospital, Tokyo 113-8677, Japan; km_statistics@tmhp.jp; 5 Department of Pathology, National Institute of Infectious Diseases, Tokyo 162-8640, Japan; tksuzuki@niid.go.jp; 6 Department of Laboratory Medicine, The Jikei University School of Medicine, Tokyo 105-8461, Japan; matsuuratomo@gmail.com; 7 Department of Infectious Disease Research, Foundation for Biomedical Research and Innovation at Kobe, Kobe 650-0047, Japan; 8 Department of Microbiology and Immunology, Hamamatsu University School of Medicine, Hamamatsu 431-3192, Japan; tesuzuki@hama-med.ac.jp; 9 Department of Neurology, PML/MS/NMO Center, Tokyo Metropolitan Komagome Hospital, Tokyo 113-8677, Japan; 10 Department of Biological Science and Technology, Tokyo University of Science, Tokyo 125-8585, Japan

**Keywords:** JC polyomavirus, pseudoviruses, PML, neutralization assay, multiple sclerosis

## Abstract

Progressive multifocal leukoencephalopathy (PML) is a demyelinating disease of the central nervous system (CNS) caused by reactivation of dormant JC polyomavirus (JCPyV). PML was mainly observed in immunocompromised individuals, such as HIV-positive patients, autoimmune disease patients, and cancer patients. Given that the presence of anti-JCPyV antibodies in serum is a risk indicator for PML development, it is essential to monitor anti-JCPyV antibody levels. In the present study, we established reporter-based single-infection neutralization assays for JCPyV and the genetically similar BK polyoma virus (BKPyV). We then confirmed the lack of cross-reactivity between the two viruses using test sera obtained from mice immunized with plasmids encoding the JCPyV or BKPyV capsid. Next, we compared neutralization antibody titers in sera from healthy donors, patients with multiple sclerosis (MS), and HIV-positive patients using an in-house enzyme-linked immunosorbent assay (ELISA) with JCPyV-like particles (virus-like particles; VLPs). A positive correlation was demonstrated between the neutralization titer (75% infectious concentration; IC75) against JCPyV and the antibody titer obtained by VLP-based JCPyV ELISA. This assay system may be applied to detect antibodies against other PyVs by generation of pseudoviruses using the respective capsid expression plasmids, and is expected to contribute to the surveillance of PyV as well as basic research on these viruses.

## 1. Introduction

Polyomaviruses (PyVs) are non-enveloped viruses with circular double-stranded DNA genomes of approximately 5.0 kb [1]. The PyV genome encodes two major regulatory proteins, the large tumor antigen (LT-ag) and the small tumor antigen, and at least two structural proteins (VP1 and VP2). LT-ag contains an origin-binding domain and a helicase/ATPase domain, representing functions that are required for viral genome replication [1]. The virion consists of 72 pentamers of the major capsid protein (VP1), with each pentamer associated with a single copy of the minor capsid protein (VP2 or VP3) [2,3]. To date, fifteen PyVs have been isolated from human specimens [4]. The first human polyomaviruses (HPyVs), BK polyomavirus (BKPyV) and JC polyomavirus (JCPyV), were identified in 1971 [5,6], with subsequently discovered HPyVs including Karolinska Institute polyomavirus (KIPyV), Washington University polyomavirus (WUPyV), and Merkel cell polyomavirus (MCPyV).

Some HPyVs have been shown to cause significant disease. Notably, in immunocompromised individuals previously infected with an HPyV, the virus can be reactivated, such that a persistent subclinical viral infection is converted to a lytic infection, resulting in viremia and potentially leading to severe or fatal diseases [7]. BKPyV causes nephropathy and hemorrhagic cystitis in kidney and in hematopoietic stem cell transplants [8]. JCPyV is a causative agent of progressive multifocal leukoencephalopathy (PML), primarily in HIV-positive patients [9]. In addition, immunomodulatory and immunosuppressive drugs for autoimmune diseases have been shown to increase the risk of PML, particularly in natalizumab-treated patients with multiple sclerosis (MS) [10]. Studies of potential risks for PML identified three risk factors: prior immunosuppressant use, duration of natalizumab treatment, and the presence of anti-JCPyV antibodies [11,12].

Detection of pathogen-specific antibodies is a prerequisite for clinical diagnosis, as well as for epidemiological studies. Regarding JCPyV antibody detection, the ELISA-based serum test for anti-JCPyV (STRATIFY JCV DxSelect) has been of limited clinical use in only natalizumab-treated patients with MS, or patients with MS considering natalizumab-treatment, but not in immunocompromised individuals with PML risks, such as HIV-positive patients and transplant recipients at the moment. In the present work, we generated reporter pseudoviruses of JCPyV and BKPyV (JCpv and BKpv, respectively) that might be useful for evaluation of the neutralizing properties of virus-specific antibodies.

## 2. Materials and Methods

### 2.1. Cell Culture

Human embryonic kidney HEK293T cells, human fetal glial SVG-A cells, and human neuroblastoma IMR-32 cells were maintained in “complete medium” consisting of Dulbecco’s Modified Eagle’s Medium (DMEM) (FUJIFILM Wako Pure Chemical Corporation, Osaka, Japan) supplemented with nonessential amino acids (NEAA) (Thermo Fisher Scientific, Waltham, MA, USA), 100 U/mL penicillin, 100 μg/mL streptomycin, and 10% fetal bovine serum. All cell culture was conducted at 37 °C in a 5% CO_2_ environment.

### 2.2. Plasmids

The reporter plasmid, pCMV Gluc dNeo, was constructed by removing the neomycin resistance-encoding gene (via flanking SmaI-SfuI restriction sites) from pCMV-Gluc (NEB, Ipswich, MA, USA). The resulting plasmid contains the cytomegalovirus (CMV) promoter; a Gaussia luciferase (GLuc)-encoding gene; and the SV40 ori, which includes the SV40 origin and enhancer elements.

Constructions of the plasmids for expression of the capsid proteins of JCPyV and BKPyV (pCAG-JCV and pCAG-BKV, respectively), and of the SV40 T antigen expression plasmid (pCI-Ts), were described previously [13].

### 2.3. DNA Immunization of Mice

Nine-week-old, female, specific pathogen-free BALB/c mice (The Jackson Laboratory Japan Inc., Kohoku, Japan) were used in the DNA immunization experiments. Intramuscular (IM) plasmid injections and subsequent in vivo electroporation were performed in groups of mice (*n* = 3 each) as described previously [14]. In brief, 50 U of hyaluronidase was first injected into each quadriceps muscle on one side of the animal. Fifteen minutes later, mice were anesthetized with isoflurane, and then 50 μg of plasmid encoding each capsid was injected into each of the same muscles of the quadriceps. An identical procedure was subsequently performed on the contralateral quadriceps muscle. Next, electric pulses were delivered to the muscles via electrode needles using an electric pulse generator (NEPA21; Nepa Gene, Ichikawa, Japan). Each injection site received three poring pulses (50 V, 30 ms) followed by three transfer pulses (20 V, 50 ms). The electroporation was repeated three times. Two to four weeks after the primary immunization, the mice received booster DNA immunizations, and serum was collected from each animal one week after the last immunization. Blood samples were collected in tubes with serum-separating medium (Bloodsepar; Immuno-Biological Laboratories, Fujioka, Japan) and centrifuged at 2500× *g* for 2 min at room temperature. The serum supernatants were collected and heat-inactivated at 56 °C for 30 min for use in the neutralization assays. The Animal Care and Use Committee of the National Institute of Infectious Diseases approved the animal experiments (Approval No. 121122), which were carried out in accordance with the approved protocol and relevant national and international guidelines.

### 2.4. Pseudovirus Production for BKPyV and JCPyV

**Figure 1:**
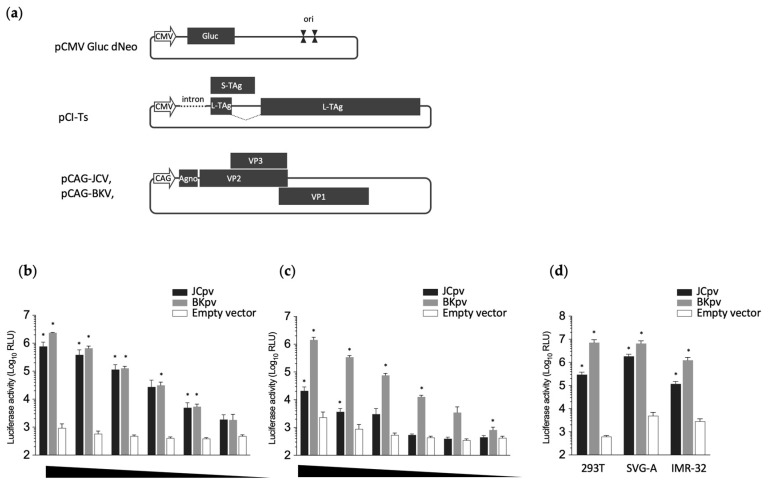
Generation of reporter pseudoviruses. (**a**) Schematic representation of the reporter plasmid (pCMV Gluc dNeo), T antigen expression plasmid (pCI-Ts), and capsid expression plasmids (pCAG-JCV and pCAG-BKV). Positions of the CMV promoter (CMV), Gaussia luciferase-encoding gene (Gluc), SV40 origin (ori), SV40 T antigen, capsid proteins (VPs) of HPyVs, and CAG promoter (CAG) are shown. To generate the pseudoviruses, HEK293T cells were cotransfected with three plasmids: pCMV Gluc dNeo, pCI-Ts, and one of the capsid expression plasmids. (**b**,**c**) Luciferase activity in HEK293T cells infected with each class of pseudovirus. Pseudoviruses, obtained as either the sonication lysate of transfected cells (**b**) or culture supernatant of transfected cells (**c**), were subjected to serial dilution and inoculated onto naïve HEK293T cells. Luciferase activity was measured in the resulting spent culture medium at 3 days post-inoculation. (**d**) The susceptibility of SVG-A or IMR-32 cells to JCpv and BKpv. The extracts from transfected cells were inoculated onto HEK293T, SVG-A, or IMR-32 cells, and luciferase activity in the resulting spent culture medium was measured at 3 days post-inoculation. The statistical significance of differences between control (empty vector) and each pv was evaluated using Student’s *t*-test (* *p* < 0.001 vs. control).

Polyoma reporter pseudoviruses were produced by transfection of HEK293T cells with three plasmids, including: pCMV Gluc dNeo, which includes a Gluc-encoding gene downstream of the CMV promoter, and which is followed by the SV40 ori (constituting a packaging signal and replication origin); pCI-Ts, an SV40 T antigen expression plasmid (promoting the replication of pCMVGluc dNeo); and an HPyV capsid protein expression plasmid (pCAG-JCV or pCAG-BKV for JCPyV or BKPyV, respectively) (Figure 1a). Briefly, HEK293T cells were grown in a 10 cm cell culture dish and cotransfected (using Polyethylenimine) with 2 μg of pCMV Gluc dNeo, 2 μg of pCI-Ts, and 10 μg of individual capsid expression plasmid. On days 1 and 2 post-transfection, the culture supernatant was replaced with fresh complete medium. The culture supernatant and cells were harvested (separately) on day 3 after transfection. The medium was filtered through a 0.45 μm syringe filter. The cells were recovered using a scraper and collected by centrifugation, then washed once with phosphate-buffered saline (PBS). The resulting cell pellet was resuspended in 4.5 mL of fresh complete medium and sonicated. The lysate then was spun down at 15,000× *g* at 4 °C for 10 min, and the resulting supernatant was used as JCpv or BKpv. For the negative control, an empty vector was used in place of the capsid expression plasmid.

### 2.5. Serum Samples

Sera from Japanese healthy donors were purchased from Clinical Trials Laboratory Services and BioIVT. Separately, serum samples from patients with MS or HIV were collected for diagnostic purposes under the auspices of protocols approved by the Ethics Committees of Tokyo Metropolitan Komagome Hospital (Approval No. 2373) and the National Institute of Infectious Diseases (Approval No. 1054). The demographics of the nonpurchased serum samples were as follows: 28 serum samples from 21 patients; donor ages between 39 and 79 years; 10 males and 11 females; 14 patients with MS, and 8 patients seropositive for HIV. All serum samples were heat-inactivated at 56 °C for 30 min prior to use in neutralization assays.

### 2.6. Neutralization Assay

Pseudoviruses (10^5^ relative light units (RLU)/well) were used for the neutralization assay. Four-fold serially diluted serum samples were mixed with pseudoviruses at a 1:1 ratio. After incubating the mixtures at 37 °C for 1 h, each serum–pseudovirus mixture was inoculated onto HEK293T cell monolayers in 96-well tissue culture plates. The plates then were incubated at 37 °C, and the culture medium was changed once daily (at 1 and 2 days post-infection). At day 3 post-infection, Gaussia luciferase activity in the supernatant was determined using the Renilla luciferase assay system (Promega, Madison, WI, USA). Each sample was assayed in quadruplicate. Luminescence was measured on a GloMax Navigator (Promega). The neutralization titers were expressed as the serum dilution producing 75% inhibition (IC75) and compared with luciferase activity obtained using a no-serum control; calculations were performed using the FORECAST function in Microsoft Excel (version 16.80; Microsoft Corporation, Redmond, WA, USA).

### 2.7. Enzyme-Linked Immunosorbent Assay (ELISA)

Details of the production and purification of VLPs were described previously [15]. Briefly, an insect cell line from Trichoplusia ni, BTI-Tn 5B1-4 (Tn5) (Thermo Fisher Scientific), was infected with recombinant baculoviruses expressing the VP1 of JCPyV- or BKPyV at a multiplicity of infection (m.o.i.) of 10 and incubated in EX-CELL 405 medium (JRH Biosciences, Lenexa, KS, USA) for 7 days at 26.5 °C. The culture medium was harvested, and intact cells, cell debris, and progeny baculoviruses were removed by centrifugation at 10,000× *g* for 90 min. The supernatant was then spun at 100,000× *g* for 1 h in a Beckman SW28 rotor. The resulting pellet was resuspended in 4.5 mL EX-CELL 405 at 4 °C overnight. After mixing with 2.1 g of CsCl, the sample was centrifuged at 100,000× *g* for 24 h at 10 °C in a Beckman SW50.1 rotor. Fraction containing VLP was harvested by puncturing the tubes with a 22-gauge needle. Aliquots (100 μL/well) of approximately 1 μg/mL VLPs in carbonate-bicarbonate buffer (Merck, Darmstadt, Germany; C3041) were adsorbed onto flat-bottom 96-well polystyrene microplates (Immulon 2; Dynex Technologies, Chantilly, VA, USA) by overnight incubation at 4 °C. An aliquot (100 μL) of 10% skim milk (SM; Difco Laboratories, Detroit, MI, USA) in PBS containing 0.05% Tween 20 (PBS-T) was added to each well, and the plates were blocked by incubation for 1 h at 37 °C. After the plates were washed three times with PBS-T, human serum samples (diluted 200-fold in 1% SM/PBS-T) were added at 100 μL/well. The plates were incubated at 37 °C for 1 h and washed three times with PBS-T. Horseradish peroxidase-conjugated goat anti-human immunoglobulin G (IgG) (Jackson ImmunoResearch, West Grove, PA, USA; 109-035-097; diluted 1:20,000 in 1% SM/PBS-T) was distributed to the plates at 100 μL/well. The plates were incubated at 37 °C for 1 h and washed four times with PBS-T. Next, a solution of substrate (ortho-phenylenediamine; Merck, C3804) and H_2_O_2_ in 0.05 M phosphate-citrate buffer (Merck, P4809) was added at 100 μL/well. The plates were incubated for 30 min in a dark room at room temperature, at which point the reactions were quenched by the addition of 4 N H_2_SO_4_ (50 μL/well). Absorbance was measured at 492 nm. All reactions were performed in duplicate.

### 2.8. Statistical Analysis

Unless noted otherwise, the error bars represent the standard deviation and are shown for experiments with *n* = 3 or greater. All between-group comparisons were carried out by two-tailed *t*-test using Prism software (version 10.1.1, GraphPad Software, Inc., Boston, MA, USA).

## 3. Results and Discussion

Polyoma reporter pseudoviruses were produced by transfection of HEK293T cells with three plasmids as described in the Materials and Methods (Figure 1a). The resulting cultures were separated into two fractions, including the spent medium (the culture supernatant of the transfected cells) or the cellular fraction (the sonication lysate of the transfected cells). These two fractions were used to inoculate naïve HEK293T cells, and luciferase activity was measured in the spent culture medium of the resulting cultures at 3 days post-inoculation.

Higher luciferase activity and lower background were seen in the supernatant of the cells inoculated with the JCpv from the cell extract (Figure 1b) than in those inoculated with the JCpv from the culture supernatant of transfected cells (Figure 1c). In contrast, elevated luciferase activity was detected in the cells inoculated with BKpv from either the cell extract or the culture supernatant of transfected cells (Figure 1b,c). This difference may reflect lower efficiency of secretion of JCPyV particles compared to BKPyV particles, at least in HEK293T cells. Luciferase activity levels in cells infected with cell lysate-derived pseudoviruses (whether JCpv or BKpv) correlated with the inoculated viral loads (Figure 1b), indicating that the luciferase assay can be used to quantify pseudovirus infection. Therefore, pseudoviruses extracted from the cells were used for subsequent experiments. We also examined the infection of SVG-A and IMR-32 cells with JCpv, as these cells are known to be JCPyV-susceptible lines that are used as models of HPyV infection of glial cells and neurons, respectively. Both cell lines showed high luciferase activity compared to the negative control (Figure 1d), indicating that JCpv possesses similar infectivity in these differing cell lines. BKpv also exhibited efficient infectivity for both SVG-A cells and IMR-32 cells.

**Figure 2:**
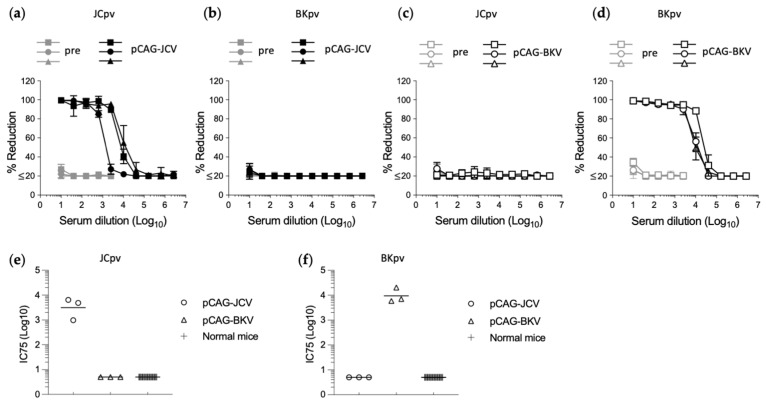
Neutralization of JVpv or BKpv by specific antiserum. Neutralization test of sera from mice (*n* = 3) immunized with the JCPyV (**a**,**b**) or BKPyV (**c**,**d**) capsid expression plasmid. Dose-dependent percentage reduction curves of each immunized mouse serum (black line) and preimmune serum (gray line) were obtained with the reporter JCpv (**a**,**c**) or BKpv (**b**,**d**), respectively. Neutralization titer shown as the inhibitory concentrations that neutralized 75% of infections (IC75) against JCpv (**e**) or BKpv (**f**) in serum from normal mice (*n* = 30) and mice immunized with the JCPyV or BKPyV capsid expression plasmid (*n* = 3). Values below the lowest dilution tested (10-fold dilution) are plotted at a value of 5 to represent less than 75% neutralization activity at the lowest dilution tested.

To examine whether infections by JCpv or BKpv were neutralized by specific anti-sera, mice (*n* = 3) were immunized by IM injection with pCAG-JCV or pCAG-BKV. Sera from immunized mice were analyzed by neutralization assays with JCpv and BKpv. All three mice immunized with pCAG-JCV showed high neutralizing activity against JCPyV (Figure 2a), and no or minimal neutralizing activity against BKpv (Figure 2b). In contrast, all three mice immunized with pCAG-BKV showed no or minimal neutralizing activity against JCpv (Figure 2c), and high neutralizing activity against BKpv (Figure 2d). To evaluate the background levels of this assay, a large number of samples from individuals with negative responses should be tested. However, it is difficult to collect a true negative human sample due to the high prevalence of antibodies to JCPyV and BKPyV in even healthy individuals. Therefore, in this study, serum from normal mice (*n* = 30) was used as a negative control for neutralization of JCpv and BKpv. The data clearly showed that all normal mice serum had no neutralizing activity against JCpv and BKpv (Figure 2e,f). These results suggested that reporter JCpv and BKpv can be used to detect specific neutralizing antibodies against each PyV with minimal cross-reactivity.

**Figure 3:**
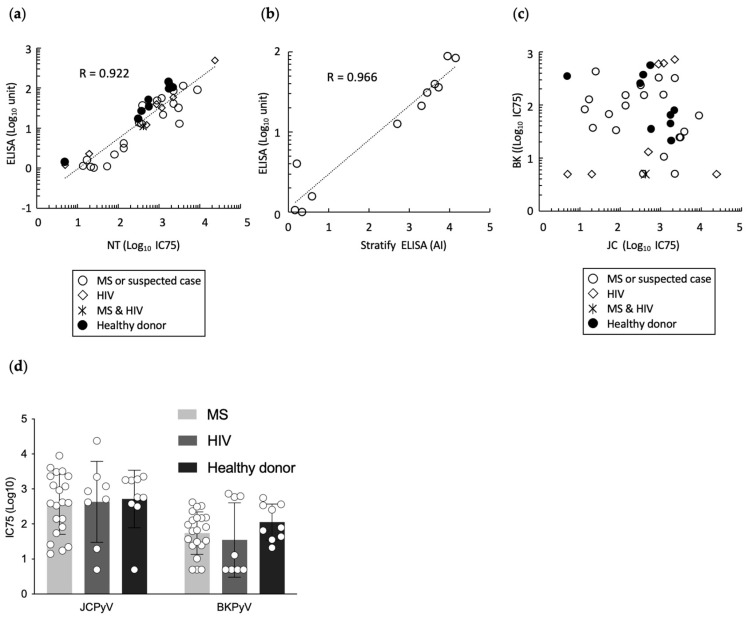
Neutralization test of serum samples from healthy donors, patients with MS, and patients with HIV. (**a**) Correlation of the neutralization titer (IC75) against JCpv (*x*-axis) with anti-JCPyV antibody titer (as assessed by VLP-based ELISA; *y*-axis) in healthy donors (●), patients with MS (○), patients with HIV (◇), and a patient with both MS and HIV (＊). The neutralization titers are shown as the inhibitory concentrations that neutralized 75% of infections (IC75). (**b**) Correlation of Antibody Index (AI) by second-generation anti-JCV antibody assay (STRATIFY JCV DxSelect) (*x*-axis) with anti-JCPyV antibody titer (as assessed by VLP-based ELISA; *y*-axis) in sera of patients with MS. (**c**) Correlation of the neutralization titer (IC75) against JCpv (*x*-axis) with IC75 against BKpv (*y*-axis) in sera from healthy donors (●), patients with MS (○), patients with HIV (◇), and patient with both MS and HIV (＊). (**d**) Neutralization titer (IC75) values against JCpv and BKpv in sera from healthy donors, patients with MS, and patients with HIV. Each open circle represents an individual IC75 value.

JCPyV causes PML under conditions of immune impairment, and an ELISA-based serum test to detect antibodies specific for JCPyV, has been used in clinical practice for PML risk stratification [16,17]. Therefore, we conducted the neutralization assay of sera from patients with MS and HIV, as well as healthy donors, using JCpv, and compared the neutralization assay antibody titers to those obtained with VLP-based ELISA [15]. This experiment employed serum samples from 9 healthy donors, 21 patients known or suspected to have MS, and 8 HIV-positive patients, including 1 HIV-positive patient with MS. Overall, a strong positive correlation (Y = −0.77411 + 0.75779 × X, R = 0.922) was detected between neutralization titer (IC75) by JCpv and antibody titer by VLP-based JCV ELISA (Figure 3a), suggesting that the neutralization assay with JCpv might serve as an alternative test to antibody measurement by ELISA. Indeed, the ELISA titer that we obtained exhibited a strong correlation (Y = 0.03911 + 0.42877X, R = 0.966) with the antibody index determined using a second-generation anti-JCV antibody assay (STRATIFY JCV DxSelect; Biogen, Cambridge, MA, USA) [17] (Figure 3b). At the same time, the IC75 values against JCpv and BKpv did not show a correlation (Figure 3c). Furthermore, the neutralizing titers against both JCPyV and BKPyV did not differ significantly among healthy donors, patients with MS, or patients with HIV by one-way analysis of variance using EZR software (version 1.61, freely available) [Figure 3d, Pr(>F) = 0.326]. Additional larger trials, along with longitudinal research, will be needed to clarify the relationship between neutralizing activity and disease condition, especially in the context of risk stratification.

In the present study, we established a neutralizing assay for multiple PyVs using reporter pseudoviruses, and demonstrated the utility of a neutralizing test for JCPyV. Although this study provided a proof of concept for the methodology to measure the neutralizing antibodies against JCPyV in human serum, optimization of experimental conditions and detailed analytical data are still needed for clinical use of the diagnostics. The following are limitations of the present study. First, we used 10^5^ RLU/well of pseudoviruses for the neutralization assay. This luciferase activity level is in the range (10^3^ to 10^6^ RLU/well) that is highly correlated with the inoculated viral loads (Figure 1b). However, the m.o.i. remains unclear because we could not detect pseudovirus-infected cells by immunostaining. Second, only a limited amount of cohort data was analyzed (Figure 3). For clinical evaluation of this assay, a large number of samples would be required from individuals with negative responses to JCPyV, such as young children, individuals previously exposed to the virus but in whom the virus is not actively replicating, and individuals in whom viral replication has recently reoccurred or has been reactivated.

A notable aspect of our neutralizing assay with reporter pseudoviruses is that it permits evaluation of functionally neutralizing antibodies, in contrast to conventional ELISA, which detects both neutralizing and non-neutralizing antibodies. Neutralization-based serology approaches are known to be superior with respect to specificity and sensitivity, while providing the ability to distinguish among different serotypes of HPyV [18]. In addition, this neutralization assay might be used to distinguish sera specific for wild-type and mutant viruses [19]. Further research will be needed to understand the significance of measuring the levels of functionally neutralizing antibodies and the association of such antibodies with pathology. Furthermore, the ELISA-based STRATIFY test has been of limited clinical use for the measurement of anti-JCPyV antibodies in only natalizumab-treated patients with MS, or patients with MS considering natalizumab-treatment. Thus, our assay may contribute to a wider range of research, such as measurement of antibody in serum and cerebrospinal fluid to examine the relationship between JCPyV and PML, in not only natalizumab-treated patients with MS, but also HIV patients with a low CD4 count with clear progression into acquired immunodeficiency syndrome. In addition, our system is expected to find application in detecting antibodies against other PyVs, given the ease of preparation of pseudoviruses simply by using a different capsid expression plasmid. Our method may also contribute to serosurveillance, as well as to basic research into PyV virology.

## Data Availability

The patient-related data are not publicly available due to privacy reasons, but are available from the corresponding author on reasonable request under the provision that data may not leave the hospital/institute premises. The plasmids and nucleotide sequence data used in this study are available from the authors upon request.
